# Cases of Injuries of the Thorax

**Published:** 1826-12

**Authors:** 


### INJURIES OF THE THORAX.

### *Cases of Injuries of the Thorax, treated at the* Middlesex Hospital.

(Continued from page 228.)

IV. *Case of Fractured Ribs, with Symptoms indicating a Collection of Air or Blood in the Cavity of the Pleura of the Side injured.* Treated by Mr. Bell.

July 31st, 1826.—William Norton, aged fifty-one, a pale and haggard-looking fellow, was brought to the hospital this day, in a state of intoxication. He had been knocked down, and several of the ribs of the left side were broken in consequence. His breathing was difficult; his pulse was hard and jerking, probably owing in part to his inebriated condition.

He was bled to ℥ xviij.; after which a bandage was applied round the thorax; and he took a dose of Calomel and Jalap.

August 1st.—He has recovered from his intoxication, and is now sensible. The bowels have not been acted upon by the medicine. The tongue is furred; pulse 100, and very hard; the respiration, although relieved, is still difficult. He complains of pain in his chest; and the sputum is streaked with blood.

He was bled to ℥ ix. with the effect of reducing the pulse; and he is to take ℥ jss. of the house-medicine every two hours, until the bowels are opened.

In the evening, the respiration became more difficult; and, although he had been twice bled so as to produce syncope, still the pulse rose again, and it was found necessary to repeat the bleeding. Notwithstanding this, however, the breathing still continued oppressed and laborious, the pulse became irregular, and, suffocation being threatened, it was thought proper to remove the bandage, which afforded him some relief. He was supported by a bed-chair; he lying upon, or rather inclining to the side affected.

From these symptoms* it was suspected that the oppression was caused either by air accumulated in the cavity of the pleura, or by the extravasation of blood. So urgent did the symptoms at length become, that it was thought necessary to send for Mr. Bell. The question of the propriety of an operation now arose, and was decided in the negative.

In his clinical observations, Mr. Bell stated his opinion to have been formed on these grounds:—1. The great difficulty and oppression of breathing appeared to indicate some defect in the opposite side, a circumstance which might render a perforation in the wounded side fatal; in illustration of which he mentioned a case of emphysema, in which the operator made his incision into the wrong side of the chest, and the immediate death of his patient was the result. 2. That, if the difficulty of breathing proceeded from air confined in the cavity, there would probably have been some accompanying emphysema. 3. If the oppression of the lungs depended upon hemorrhage into the thorax, an opening, unless very considerable, would evacuate the serum only, but not the coagulum. He then made some remarks on the very common error of making openings into the cavities of the body for the discharge of extravasated blood; practitioners forgetting that, when blood escaped from a vessel, it no longer remained fluid, but immediately coagulated. It is, therefore,

* The sudden and great oppression of the breathing, and the irregular pulse, gave one an idea that a collection of air or blood in the injured cavity might be the cause of such threatening symptoms: this led to a more minute examination. The whole parietes of the chest appeared to have much less motion than is usually observed when the respiration is excited. The left (the side affected) was evidently more protuberant than the right; and, on making an examination, the former was found to be an inch larger than the latter. *The man was right-handed,* and said that he did not remember having had any disease of the chest. The house-surgeon applied the stethoscope: the respiratory murmur on the left side was hardly perceptible, whilst on the right side it was distinctly heard. The next day, the respiration could not be detected on the injured side; but on the opposite side it was as audible as on the preceding day.

by no means so easy to evacuate the extravasated blood as might be at first supposed; and he gave as an example the practice which had been injudiciously recommended, of opening the dura mater for the discharge of effused blood.

Mr. Bell, in his clinical lecture on this case, took occasion to refer to the structure of the thorax; showed how the centre of motion could be changed by the change of position; and that the side of the thorax hurt was made the fixed point by lying on it, so that the other side might be more freely dilated. Had the patient lain on the sound side, then the motion of both would have been impeded,—the one by the hurt, the other by the position.

August 2d.—Respiration is more easily performed; the other symptoms continue much the same. He has some cough, for which a demulcent mixture, with compound tincture of camphor, was ordered to be taken three times a-day. The bowels have been freely opened, and the pulse has lost some of its hardness, but is still irregular.

3d.—Little alteration in the symptoms till towards evening, when his breathing again became more difficult; and, as the pulse had also acquired more strength, general blood-letting, to the amount of sixteen ounces, was once more had recourse to. The blood has been cupped and buffed throughout: in that taken to-day, the crassamentum was more easily broken down, and the serum was in greater quantity.

A draught, consisting of Acetate of Ammonia, Tartarised Antimony, and Compound Tincture of Camphor, three times a-day.

4th.—The respiration is performed with much more ease; pulse eighty-eight, and a great deal softer. He is completely blanched, and has a truly ghastly appearance.

5th.—Much better. The bandage reapplied, from which he has experienced great relief.

14th.—To-day he had an attack of fever, accompanied by violent pain in the head and some degree of delirium; pulse quick; tongue dry and furred.

Eight ounces of blood were taken from the arm, with manifest relief to the symptoms; and he was ordered to continue the Antimonial Mixture, with the omission of the Compound Tincture of Camphor.

16th.—All the symptoms of fever have subsided.

19th.—Discharged cured.

V. *Case of Fracture of the Sternum.* Treated by Mr. Joberns.

Dennis Pade, aged thirty-five, was admitted into the hospital, June 26th, 1826, in consequence of an injury which he had received by falling from a hayrick across the shaft of a cart, a height of about fifteen feet. It appears that he fell with the whole weight of his body upon the sternum and anterior part of the chest, and that he was thus as it were doubled over the shaft.

He was insensible for some time after the fall. When brought to the hospital, he had somewhat recovered, and could relate many of the circumstances connected with the accident. His breathing was difficult and constrained, and he complained of much pain in the upper part of the sternum. He spoke in a whisper, and appeared to be fearful of raising his voice.*

On examination, there was found to be a transverse fracture of the upper part of the sternum, near the junction of the triangular and middle portions of that bone. The inferior division of the fractured bone was considerably elevated above the level of the superior, and appeared in some degree to overlap it. Efforts were made to replace the fractured bone, by putting pillows under the back, and by pressing on the elevated portions during deep inspirations: any violent measures, however, were carefully avoided. These endeavours proving ineffectual, further attempts at reduction were relinquished. An examination of the ribs was made, but no injury could be detected.

He complained of a grating sensation, accompanied by pain in the situation of the fracture. The pulse was full and strong. He was bled largely, and until syncope was produced. The chest was effectually fixed by the application of a bandage, and he was supported in the half-erect posture by a bed-chair.

The bowels were well opened with the house-medicine; and he was ordered to take a draught three times a-day, containing the Acetate of Ammonia and Tartarised Antimony, but so administered as to avoid sickness.

No unfavourable symptoms occurred until the tenth day after his admission: he then complained of increased pain in his chest, and he had a troublesome cough; the pulse was full and accelerated. He was again bled until an evident impression was made on the vascular system. This bleeding effectually relieved him.

He continued to take the above draught, with the addition of half a drachm of the Compound Tincture of Camphor.

From this time he gradually improved; the grating sensation experienced on coughing diminished daily, and, on the 7th of August, there was no pain in the seat of the fracture. He complained only of an uneasy feeling near the insertion of the sixth rib into its cartilage. He left the hospital August 22d.

In reading this case at the clinical lecture, Mr. Bell remarked, with respect to the attempts to reduce the fractured portions to their natural position, that there was very considerable danger in this practice of wounding the pericardium, or even the cava or sinus. In this very case, he said, we saw that this reduction was not necessary to recovery, since the bones had consolidated, although the one portion lay over the other.

* This symptom was observed in all the cases of injuries of the thorax, and, although apparently trivial, becomes of importance from its very constant occurrence.

VI. *Case of Fracture of the Sternum.* Treated by Mr. Shaw.

Joseph Beeson, aged twenty-six, a fine and powerful young man, was admitted into the hospital August 21st, 1826. He had been attempting to move an iron plate of seven hundred weight, which rested against a wall. The weight of it overbalanced him, and he fell with this great mass of iron upon him. Two Irishmen, who saw the accident, immediately extricated him from this perilous situation.

His breathing was quick and constrained, and appeared to be performed with pain: he was evidently afraid of expanding his chest, and he spoke in a low tone of voice. About an inch and a half of the upper part of the sternum had been broken transversely from the body of the bone. A crepitus might be felt in the situation of the fracture, either by pressing on the ribs or the sternum, and he complained of great pain in this part. His pulse was full and strong.

Twenty-five ounces of blood were taken from the arm: this afforded him very great relief. A bandage was applied round the chest, and he was supported by a bed-chair. He was well purged with Calomel and Jalap; after which he took a draught three times a-day, composed of Tartrate of Potass and Tartarised Antimony.

The large and efficient blood-letting at first employed appeared to have rendered any repetition of it unnecessary. The case proceeded so favourably, the patient felt himself so well, and expressed so much anxiety to return to his family, that he was allowed to leave the hospital on the 12th of September; with injunctions, however, not to return to his work (which was laborious) for some weeks.

We have since seen him, and he has almost acquired his former strength. If he attempts, however, to lift a heavy weight, he still feels weak at the chest.

VII. *Case of Fracture of the Sternum, with Wound of the Heart,* brought to the hospital January 23d, 1826.

Richard Carpenter had been employed in repairing the third story of a house: in jumping from the window to the scaffolding erected before the house, he sprung with such force that he was unable to preserve his footing, and he was precipitated forwards into the street. He was found lying flat on his face; life was quite extinct.

*Examination.*—On dissection, the sternum was seen to be fractured in three places transversely: the fracture in the centre of the bone was the most complete; the broken extremities had been thrust in upon the cavity of the chest, and had torn both the pericardium and heart. The pericardium and anterior mediastinum were filled with blood. There was a rent in the right ventricle of the heart, which was rather more than an inch in extent. The heart was contracted, and empty.

It is to be observed, that, although the wound of the heart in its contracted state measured only an inch, yet, after the fibres of the ventricle had been relaxed by maceration in water for a day, the same rent was four inches in length.* It extended from about an inch from the root of the pulmonic artery, in a semi-circular form, to the apex of the heart, thus laying open the whole cavity of the right ventricle.

His spine was also fractured through the bodies of the tenth dorsal and first and second lumbar vertebræ.

### VIII. *Case of Fracture of the Sternum, where the Patient died from internal Hemorrhage.* Treated by Mr. Shaw.

John Tipling, a coachman, about thirty-five years of age, was brought to the hospital on the evening of July 8th, 1826. He had been thrown from his coach-box, while driving very rapidly, and the wheel of the carriage had passed over his chest. His fellow servants state that he remained for some time insensible, during the continuance of which state he had been bled by a medical man.

He was quite sensible when brought to the hospital, and said that all his pain was in the chest; the integuments of which were black, and so much swollen, as to render an accurate examination of the sternum and anterior parts of the thorax next to impossible. On pressing on the fore part of the chest, however, a crepitus was felt; and, on passing the hand deep into the axilla, and then pressing on the ribs, the second and third yielded, and appeared to be broken near the sternum. The clavicle on the same side was loose at its sternal extremity, and seemed as if fractured at this part; but the swelling of the coverings of the chest, from extravasated blood, was so great, that these opinions could be only conjectural. His pulse was small and weak, and his respiration was somewhat oppressed. He was ordered to be kept perfectly quiet.

On visiting him about an hour and a half afterwards, we found him lying on his face. His breathing was so gentle as to be scarcely perceptible; his countenance was blanched; his pulse could hardly be felt, and he lay quite insensible. In short, he appeared to be in a dying state, and, from the symptoms present, we were led to conclude that he was sinking from internal hemorrhage.

A drachm of the Aromatic Spirit of Ammonia, in Camphor Mixture, was given him.

In half an hour, the pulse could not be felt, and the surface was cold; the continuance of respiration was the only indication of life

* The appearance presented by the wound of the heart in this case, brings to our recollection similar appearances in a wound of the heart caused by a pistol bullet. Although the ball was so small as to belong to a pocket-pistol, the rents in both ventricles were very extensive, and of size sufficient to admit the points of three fingers. This apparent anomaly is to be explained by the action of the heart as a muscular organ, and is similar to what occurs in wounds of muscles in other parts of the body. It is a fact of the utmost importance as applied to the bladder, where the lithotomist, cutting into an empty and contracted bladder, may make an incision of frightful extent before he is enabled to extract the stone.

Brandy was now administered. About an hour after this, the pulse became again perceptible, and, gradually rising, he shortly afterwards became sensible. The brandy was then discontinued.

He remained perfectly sensible during the night: he was sitting up in his bed and talking with his friends in the morning, and appeared to be quite recruited. Whilst talking with them, he suddenly fell back on his pillow, as they imagined in a fit. The house surgeon was immediately called: he found the patient drawing extremely slow and convulsive inspirations, foaming at the mouth, and quite senseless; the pulse at the wrist was no longer to be felt. He died in a few minutes.

*Dissection.*—The integuments covering the anterior parts of the chest were so much swollen with extravasated blood, as to measure nearly three inches in thickness. The second and third ribs, with their cartilages, were detached from their sternal articulations; the second was also fractured near the union with its cartilage. The part of the sternum which is connected with the right clavicle was broken off from the rest of the bone, and presented at its lower part a sharp and angular point.

The sternum being raised, a large quantity of coagulated blood was discovered gorging the cellular structure of the anterior mediastinum. The hand was easily passed along the course of the large venous trunks to the right side of the neck, the whole of which track was stuffed with coagula of blood. There was a large pouch situated deep in the neck and by the side of the vertebræ, which was also full of clotted blood.

The pericardium, heart, and great vessels of the chest, appeared to be entire. The wounded vessel was not discovered. It is probable, however, that it was but of small size, or the patient would not have survived so long.

The suddenness of this man's death suggested an examination of the spine. Three of the spinous processes of the upper dorsal vertebræ were fractured, but the vertebral canal was entire.

The fracture of the sternum has ever been considered as an injury attended with the greatest danger; insomuch that some have ranked it as next in importance to injuries of the brain and spinal marrow. That such an opinion should have at all times obtained is by no means extraordinary, when the situation, connexion, and office of this bone are considered.

The connexion of this bone with the ribs through the medium of the elastic cartilages, affords to it not only a means of support, but also of defence. "The sternum stands so much exposed that, did we not naturally guard it with our hands, fractures must be very frequent."

Thus it is found that, when this fracture does occur, it has generally been produced by some great force,—as by a cart-wheel or some other heavy body passing over the chest, or by some means equally violent. It is more than probable, then, that the danger attending the fracture of the sternum arises, in a great measure, from the causes by which it is so often produced; by which means the important viscera of the thorax become so much injured as to render this fracture very frequently fatal. And it is not from the supervention of inflammation that the patient generally dies, for over this we have some control; but it is from the immediate effects of the accident—the internal hemorrhage.

### IX. *Case of Dislocation of the Ribs from their Cartilages.* Treated by Mr. Bell.

John Bean, aged seventy-two, was admitted on the 4th of June, 1826, a month after the occurrence of the accident, which happened in the following way:

He had been standing up in his cart whilst driving it down a hill; an inequality in the road producing a sudden jerk, threw him forwards, and he fell with his breast on the shaft, striking the fore part of the chest with much violence. So great was the injury thus produced, that it was found necessary to have him conveyed to his home, where he was attended by a surgeon, who bled him, and applied a bandage to the thorax. Directions were given that he should be kept in a state of perfect quietude; and, from his own account, the injury appears to have been followed by no unfavourable symptoms.

It was now found that the sternum, and all the cartilages of the ribs, have been forced in upon the cavity of the thorax, and that a dislocation, or rather a diastasis, of these cartilages from their ribs has been the consequence.

The whole contour of the chest presented a most extraordinary appearance, but to the touch it conveyed a well-known sensation. We recognised something at once familiar: we know not how to describe it better, or how to furnish an illustration more applicable, than that given by Mr. Bell in a similar case, in his "Clinical Surgery."* He says that "his chest was like that of a dead body in which the thorax had been opened, and the sternum left loose under the integuments; for, on both sides, the end of each rib, at its junction with the cartilage, stood out distinctly marked and prominent."

The sternum and its cartilages still remain moveable, and produce (according to his own expression) a great weakness of the chest: this and his long confinement to bed have rendered him extremely feeble, and are the reasons why he seeks relief at this hospital.

He has a slight difficulty in breathing and a tickling cough, but there is no pain in the chest. The bowels are free, and the tongue is clean.

* Page 172.

**He is to take a Demulcent Draught, with fifteen minims of the Compound Tincture of Camphor; and the chest is to be swathed with a bandage.**

He progressively improved. The sternum and cartilages of the ribs remained depressed, but their attachments to the ribs became more firm. He was discharged on the 18th of July, having received much benefit.

Mr. Bell, in his clinical lecture, after admiring the powers of nature displayed in this and some cases of a similar kind, remarked on the necessity of confining the chest by bandage, to restrain the motion of the fractured bones, that, although in most cases no bad consequences resulted from the neglect of bandaging, yet it gave unspeakable relief, and it prevented caries. He gave an instance where, in consequence of the fracture of three ribs, caries of their extremities had taken place, and suppuration of the pericardium contiguous to these portions of diseased bone.

In speaking of bandaging the chest to suppress the motions of the ribs or sternum, he drew our attention to the case of Norton, (No. 4,) where, in the first attempt to swathe the body, it was attended with intolerable oppression, and yet at an after period it was followed with remarkable relief. Respiration and circulation, he said, were two parts of one function; they must always correspond. If we suppress the action of the ribs, and leave only the diaphragm free, we limit the play of the lungs to one half; and if, while we thus impede the action of the lungs, the heart and arteries remain full of blood and active, the patient becomes suffocated! But when we have bled him, and have reduced the activity of the circulating system, we may then limit the motions of the chest. We observe then, he said, the necessary connexion betwixt the application of the roller on the chest and the use of the lancet. Without attention to this, the unfortunate man is left to unutterable agony,—perhaps to suffocation. In these cases we bleed for two reasons: first, to avoid inflammation; and secondly, to permit the thorax to be bound.

### X. *Case of Wound of the Lungs, which was followed by extensive Suppuration.* Treated by Mr. Bell.

Thomas Hyde, aged seventeen, admitted October 28th, 1826. This lad, about twelve weeks ago, fell from the top of a wheat-stack, twenty feet high, upon a wooden paling, one of the sharp staves of which was forced in behind the right clavicle, and entered the upper part of the chest. A piece of wood, five inches in length and one and a half in breadth, was broken off abruptly in the wound, where it remained until the assistance of Mr. Brown, of Epsom, was procured, who found it necessary to employ considerable force to extract the splinter. The wound made by it readily admitted of the introduction of three fingers, which might be passed their whole length into the chest. Much air and blood escaped. As soon as the bleeding ceased, the edges of the wound were retained in contact by adhesive straps. His breathing was very difficult, insomuch that it was found necessary to bleed him twice the first night. He was bled the three following days, and the antiphlogistic treatment was followed up in all its details. This had the effect of preventing the occurrence of high inflammatory action; but adhesion in the external wound did not take place.

In eight or nine days the respiration became much easier; and, about the twelfth day after the accident, as his mother informed us, "the breath ceased to come through the wound;" since which time large quantities of very offensive pus have been discharged through the wound, the quantity of matter discharged frequently amounting to half a pint daily. About the third week he began to cough: this cough has latterly become very troublesome, and has been accompanied by much expectoration.

He is now extremely emaciated, and resembles a person in the last stage of pulmonary phthisis, although previously to the accident he was a stout and healthy lad. He can however stand, and even walk a few steps without assistance: this he could not do three weeks ago; indeed, he was then so weak as to be unable to sit up in bed. This improvement his mother attributes to a change in his diet; for latterly he has been allowed nourishing food, as eggs, &c. and occasionally a mutton-chop.

There is at present but little discharge from the wound: it has been gradually diminishing in quantity for the last fortnight. The pus, however, which is discharged is of a scrofulous character. He has a troublesome cough, and he expectorates a yellow mucus, mixed with pus. Pulse 111, small and weak. He had a diarrhœa a week ago, and he has been subject to profuse perspirations at night. These unfavourable symptoms have now subsided.

The right side of the chest measures an inch less in circumference than the left. The spinous processes of the middle dorsal vertebræ incline towards the left side, and might lead to the idea of there being a lateral curvature dependent upon the disease of the chest, as described by Laennec; but it appears to be rather an irregular formation or disposition of the spinous processes.

Mediate auscultation discovers that the respiration is entirely deficient in the upper and anterior part of the right side of the chest, in the neighbourhood of the wound. It is extremely indistinct in the lower and anterior part of the same side; but the respiratory sound is very perceptible in the posterior part of the cavity, although it is even here less distinct than on the opposite side. The heart pulsates strongly, and its apex is situated more to the left side than usual.

He is to take the following draught three or four times a-day:—Inf. Lini ℥ ij.; Træ. Opii Camp. ʒ ss.; Vin. Ipecac. gtt. xx.—His diet is to consist of light but nutritious food, avoiding at the same time all such things as may prove stimulating.

November 13th.—Continues to improve. His expectoration becomes less puriform in appearance, and is much diminished in quantity; his cough is not so troublesome, and his sleep is consequently less disturbed. The wound is healed.

23d.—This patient may now be regarded as convalescent; should any unfavourable change occur we shall mention it in a future number.

